# Preoperative serum thyroglobulin predicts initial distant metastasis in patients with differentiated thyroid cancer

**DOI:** 10.1038/s41598-017-17176-6

**Published:** 2017-12-05

**Authors:** Hosu Kim, Young Nam Kim, Hye In Kim, So Young Park, Jun-Ho Choe, Jung-Han Kim, Jee Soo Kim, Jae Hoon Chung, Tae Hyuk Kim, Sun Wook Kim

**Affiliations:** 1 Division of Endocrinology & Metabolism, Department of Medicine, Thyroid Center, Samsung Medical Center, Sungkyunkwan University School of Medicine, Seoul, Korea; 2 Division of Endocrinology, Department of Medicine, Gyeongsang National University Changwon Hospital, Changwon, Korea; 3 Division of Breast and Endocrine Surgery, Department of Surgery, Samsung Medical Center, Sungkyunkwan University School of Medicine, Seoul, Korea

## Abstract

Differentiated thyroid cancer (DTC) generally has a favorable prognosis. However, a small percentage of patients suffer from initial distant metastasis (DM). To date, there is no effective predictor for the presence of initial DM. The aim of this study was to determine if preoperative serum thyroglobulin (Tg) level could predict initial DM in DTC. We reviewed an institutional thyroid cancer database from October 1994 to February 2016. To determine the Tg cutoff for predicting initial DM, 4,735 patients who were diagnosed with DTC were included in this study. Fifty-seven patients (1.2%) were identified as having DTC with initial DM. Median preoperative Tg level was 328.4 ng/ml in the initial DM group and 10.0 ng/ml in the non-DM group. Initial DM was the most important factor affecting serum Tg level (*β* = 2,049.32 ± 103.40; *P* < 0.001). The Tg cutoff level that distinguished overall DM with the greatest accuracy was 63.4 ng/ml [area under the ROC curve 0.914, sensitivity 84.2%, specificity 90.6%, negative likelihood ratio (LR) 0.17, and positive LR 8.97]. Preoperative Tg levels were useful for predicting initial DM of DTC. Measurement of serum Tg in patients with DTC may guide preoperative staging evaluation and initial treatment.

## Introduction

Most patients with differentiated thyroid cancer (DTC) have a favorable prognosis and initial distant metastasis (DM) is rare^1,2^. However, the mortality rate significantly increases when initial DM is present^3–7^. Therefore, early diagnosis and treatment of initial DM is important for the treatment of DTC patients^8,9^. For medullary thyroid carcinoma, serum calcitonin levels predict initial DM, recurrence and prognosis, but DTC has no available predictor^10,11^.

Thyroglobulin (Tg) is a glycoprotein that is secreted from follicular cells of the thyroid gland. Therefore Tg reflects the burden of the follicular cell, whether it is benign or malignant^12^. Tg helps predict DTC recurrence in a postoperative setting, but its usefulness for preoperative diagnosis is less clear^13–19^. Tg levels can rise when follicular cells increase, such as in goiter and thyroiditis, even if they are not indicative of pathological status^20^. However, in patients with a high tumor burden, such as patients with initial DM, serum Tg concentration may increase unequivocally^8,21–23^.

The purpose of this study was to determine the correlation between preoperative serum Tg concentration and the existence of distant metastasis in DTC patients. We evaluated whether preoperative serum Tg concentration could be used as a predictive marker for initial DM in clinical settings.

## Results

### Differences between presence of initial DM and non-DM group

Of the 4,735 enrolled patients, 57 had initial DM. No difference was observed in age and sex between the initial DM and non-DM group. By tumor histology, follicular thyroid carcinoma (FTC) was more frequent in the initial DM group than the non-DM group (35.1% vs. 2.0%, P < 0.001). Mean tumor size was 3.19 cm in the initial DM group, which was larger than the non-DM group. However, multifocal tumor burden was not significantly different between the two groups. Gross extrathyroidal extension (ETE), lymphatic invasion, blood vessel invasion, positive resection margin and lymph node metastasis (LNM) > 5 were all significantly higher in the initial DM group than the non-DM group (Table 1).

**Table 1:** Differences between initial DM and non-DM groups.

Characteristics	Initial DM group (*n* = 57)	Non-DM group (*n* = 4678)	*P* value
Age at diagnosis (years)	47.75 ± 17.58	46.36 ± 11.24	0.555
Sex (female)	42 (73.7%)	3670 (78.5%)	0.417
RAI treatment	55 (96.5%)	3003 (64.2%)	**<0.001**
Initial CND	44 (77.2%)	3992 (85.3%)	0.091
Tumor histology			**<0.001**
PTC	37 (64.9%)	4585 (98.0%)	
FTC	20 (35.1%)	93 (2.0%)	
Tumor size (cm)	3.19 ± 1.94	1.04 ± 1.02	**<0.001**
Multifocal tumor (tumor number)	1.57 ± 1.05	1.51 ± 1.01	0.633
Positive gross ETE	19 (33.3%)	568 (12.1%)	**<0.001**
Positive resection margin	13 (22.8%)	211 (4.5%)	**<0.001**
LNM > 5	26 (54.6%)	503 (10.8%)	**<0.001**
Positive lymphatic invasion	4 (7.0%)	57 (1.2%)	**0.006**
Positive blood vessel invasion	17 (29.8%)	75 (1.6%)	**<0.001**
Preoperative Tg concentration (ng/ml)	328.4 (2.3–42680.0)	10.0 (0.1–6542.0)	**0.006**

Continuous data were given as mean ± SD; categorical data were given as absolute numbers (percentage). Preoperative Tg concentration was presented as median (range). χ2-test or Fisher’s exact test were used for categorical data, and t-tests or the Mann-Whitney test for continuous data. DM, distant metastases; RAI, radioactive iodine; CND, central neck dissection; PTC, papillary thyroid carcinoma; FTC, follicular thyroid carcinoma; ETE, extrathyroidal extension; LNM, lymph node metastases; Tg, thyroglobulin.

### Characteristics of patients with initial distant metastasis

**Figure 1:**
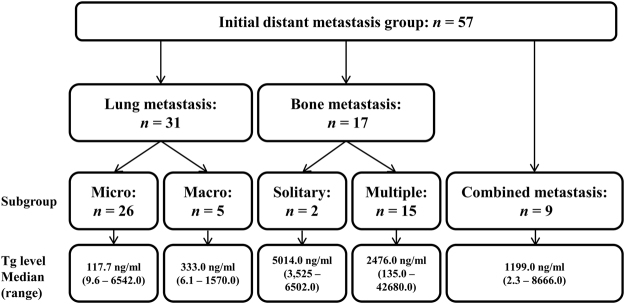
Flow diagram of the study. Fifty-seven patients were diagnosed with initial distant metastasis (DM). Patients were classified according to site of distant metastasis. Diagnoses were 31 with lung metastasis, 17 with bone metastasis, and 9 with combined lung/bone metastasis. Lung metastasis were subdivided into micronodular and macronodular. Bone metastasis were subdivided into solitary and multiple.

Overall, 57 patients were diagnosed with initial DM: 37 with papillary thyroid carcinoma (PTC) and 20 with FTC. In addition, 26 patients (45.6%) were classified as having micronodular lung metastasis, and 5 (8.8%) with macronodular lung metastasis; two patients (3.5%) were classified as having single bone metastasis, and 15 (26.3%) with multiple bone metastases. Nine patients (15.8%) had combined lung and bone metastasis. Most lung metastasis were PTC (*n* = 29), with two that were FTC. Also, 13 FTC patients were diagnosed as having bone metastasis and 4 were diagnosed as having bone metastasis from PTC (Fig. 1).

Micronodular lung metastasis was associated with the lowest preoperative serum Tg, with a median of 117.7 ng/ml (range 9.6–6,542.0 ng/ml) among initial DM subgroups. Macronodular lung metastasis had a preoperative serum Tg median of 333.0 ng/ml (range 6.1–1,570.0 ng/ml). Preoperative serum Tg was significantly higher when bone metastasis was present. Preoperative serum Tg for solitary bone metastasis had a median of 5,014.0 ng/ml (range 3,525.0–6,502.0 ng/ml), multiple bone metastasis had a median of 2,476.0 ng/ml (range 135.0–42,680.0 ng/ml), and combined metastasis had a median of 1,199.0 ng/ml (range 2.3–8,666.0 ng/ml) (Fig. 1).

### Factors associated with preoperative serum Tg elevation

Preoperative serum Tg concentration was higher in the initial DM group than the non-DM group. Tg concentration for the initial DM group was a median of 328.4 ng/ml (range 2.3–42,680.0 ng/ml) compared to the non-DM group with a median of 10.0 ng/ml (range 0.1–6,542.0 ng/ml) (Table 1). Preoperative serum Tg concentration for patients with FTC was significantly higher than for patients with PTC (median 87.6 ng/ml, range 0.5–42,680.0 for FTC and 9.9 ng/ml, range 0.1–8666.0 for PTC, *P* < 0.001). Preoperative serum Tg concentration was higher for large tumors (β = 12.20, *P* < 0.001) and blood vessel invasion (median 51.9 ng/ml, range 0.8–22,666.0 for positive blood vessel invasion and median 10.0 ng/ml, range 0.1–42,680.0 for negative blood vessel invasion). Multivariate linear regression analysis showed only FTC, large tumor size, blood vessel invasion, LNM > 5 and initial DM were significantly associated with preoperative serum Tg concentration (*β* = 662.93 and *P* < 0.001 for FTC, *β* = 48.82 and *P* < 0.001 for tumor size, *β* = 378.59 and *P* < 0.001 for blood vessel invasion, *β* = −70.19 and *P* = 0.045 for LNM > 5, *β* = 2049.32 and *P* < 0.001 for initial DM) (Table 2).

**Table 2:** Factors associated with preoperative serum Tg elevation.

Characteristics	Unadjusted		Adjusted	
	*β* ± SE	*P* value	*β* ± SE	*P* value
Age at diagnosis (years)	0.75 ± 1.02	0.460	—	—
Sex (female)	11.69 ± 28.09	0.677	—	—
Tumor histology (FTC)	1253.39 ± 73.52	**<0.001**	662.93 ± 80.13	**<0.001**
Tumor size	12.20 ± 10.71	**<0.001**	48.82 ± 10.86	**<0.001**
Multifocal tumor (tumor number)	−6.12 ± 11.40	0.591	—	—
Positive lymphatic invasion	74.57 ± 102.51	0.467	—	—
Positive blood vessel invasion	1135.89 ± 82.11	**<0.001**	378.59 ± 86.97	**<0.001**
Positive resection margin	59.62 ± 54.45	0.274	—	—
LNM > 5	39.74 ± 36.69	0.279	−70.19 ± 35.04	**0.045**
Positive gross ETE	19.97 ± 35.08	0.569	—	—
Presence of initial DM	2456.00 ± 99.82	**<0.001**	2049.32 ± 103.40	**<0.001**

Linear regression analysis was performed. SE, standard error; FTC, follicular thyroid carcinoma; ETE, extrathyroidal extension; LNM, lymph node metastases; DM, distant metastases.

### ROC analysis for identifying initial distant metastasis

**Figure 2:**
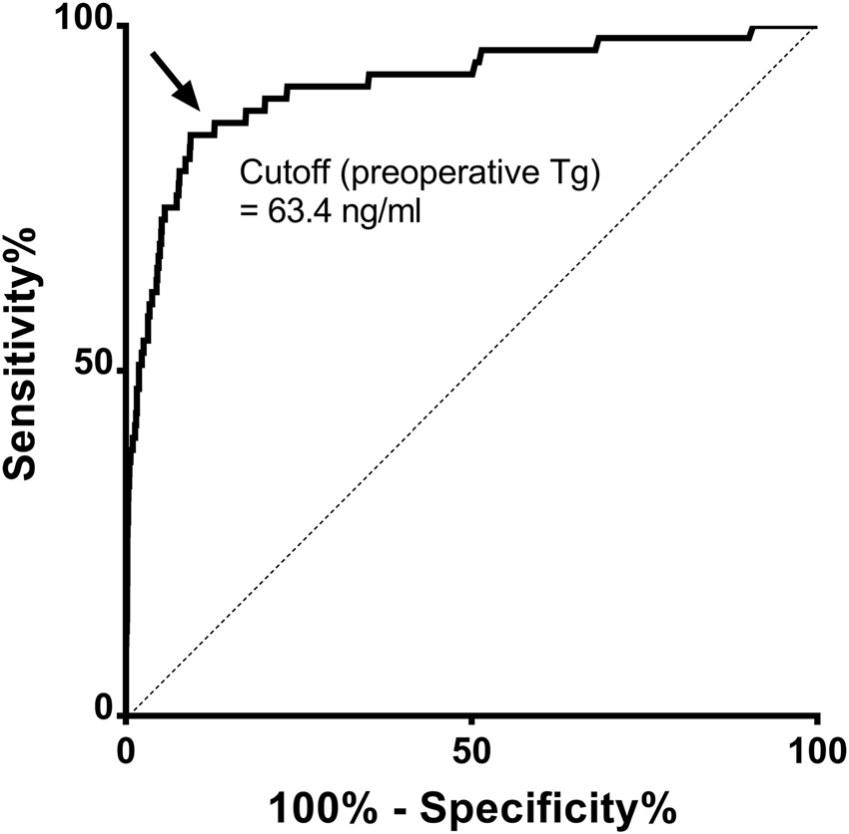
ROC curves for differentiating between the presence of initial distant metastases and non-distant metastases groups. Area under the ROC curve (preoperative serum Tg), 0.914 (95% CI, 0.869–0.960). Cutoff (preoperative serum Tg), 63.4 ng/ml; sensitivity, 84.2%; specificity, 90.6%; positive LR, 8.97; negative LR, 0.17; PPV, 9.3%; NPV, 96.4%.

In ROC analysis, the optimal cutoff for predicting overall DM was 63.4 ng/ml with area under the ROC curve of 0.914 and corresponding sensitivity 84.2%, specificity 90.6%, negative likelihood ratios (LR) 0.17, and positive LR 8.97 (Fig. 2). Negative predictive value (NPV) was 96.4% and positive predictive value (PPV) was 9.29% in our cohort. When study populations were stratified by tumor histology, the optimal cutoff for PTC to predict initial DM was 63.4 ng/ml and 1192.0 ng/ml for FTC (sensitivity 75.7%, specificity 91.4% for PTC; sensitivity 75.0% and specificity 92.5% for FTC).

### Univariate and multivariate logistic regression analyses

Clinical characteristics including preoperative serum Tg (>63.4 ng/ml or≤63.4 ng/ml), age at diagnosis, sex (male or female), tumor size, multifocal tumor, lymphatic invasion, blood vessel invasion, positive resection margin, LNM > 5 and presence of gross ETE were analyzed as independent variables using logistic regression analyses (Table 3). In univariate logistic regression analyses, preoperative serum Tg > 63.4 (odds ratio [OR], 51.50; 95% confidence interval [CI], 25.10–105.66; *P* < 0.001), tumor size (OR, 1.90; 95% CI, 1.66–2.17; *P* < 0.001), lymphatic invasion (OR, 6.12; 95% CI, 2.14–17.47; *P* = 0.001), blood vessel invasion (OR, 26.08; 95% CI, 14.05–48.08; *P* < 0.001), positive resection margin (OR, 6.26; 95% CI, 3.32–11.79; *P* < 0.001), LNM > 5 (OR, 6.69; 95% CI, 4.10–11.82; *P* < 0.001), and presence of gross ETE (OR, 3.62; 95% CI, 2.07–6.32; *P* < 0.001) were significant factors that distinguished between the initial DM and non-DM groups. In multivariate logistic regression analyses, preoperative serum Tg (OR, 24.62; 95% CI, 11.59–52.28; *P* < 0.001), age at diagnosis (OR, 1.03; 95% CI, 1.01–1.05; *P* = 0.015), tumor size (OR, 1.11; 95% CI, 1.08–1.30; *P* < 0.001), blood vessel invasion (OR, 8.06; 95% CI, 3.78–17.17; *P* < 0.001), positive resection margin (OR, 4.01; 95% CI, 1.84–8.76; P < 0.001), and LNM > 5 (OR, 4.08; 95% CI, 2.14–7.79; *P* < 0.001) were independent predictive factors for differentiating between the initial DM and non-DM groups.

**Table 3:** Logistic regression analyses of initial distant metastases according to clinicopathological factors.

Characteristics	Unadjusted		Adjusted	
	Odds ratio (95% CI)	*P* value	Odds ratio (95% CI)	*P* value
Age at diagnosis (years)	1.01 (0.99–1.03)	0.359	1.03 (1.01–1.05)	**0.015**
Sex (female)	0.77 (0.43–1.39)	0.386		
Tumor histology (FTC)	26.65 (14.90–47.66)	**<0.001**		
Tumor size	1.90 (1.66–2.17)	**<0.001**	1.11 (1.08–1.30)	**<0.001**
Multifocal tumor(tumor number)	1.06 (0.84–1.33)	0.633		
Positive lymphatic invasion	6.12 (2.14–17.47)	**0.001**		
Positive blood vessel invasion	26.08 (14.05–48.08)	**<0.001**	8.06 (3.78–17.17)	**<0.001**
Positive resection margin	6.26 (3.32–11.79)	**<0.001**	4.01 (1.84–8.76)	**<0.001**
LNM > 5	6.69 (4.10–11.82)	**<0.001**	4.08 (2.14–7.79)	**<0.001**
Positive gross ETE	3.62 (2.07–6.32)	**<0.001**		
Tg 63.4	51.50 (25.10–105.66)	**<0.001**	24.62 (11.59–52.28)	**<0.001**

Logistic regression analysis was performed. CI, confidence interval; FTC, follicular thyroid carcinoma; ETE, extra-thyroidal extension; LNM, lymph node metastases; Tg, thyroglobulin.

## Discussion

This study demonstrated that preoperative serum Tg concentration well predicted initial DM in DTC patients. We also proposed a specific Tg cutoff value of 63.4 ng/ml as an optimal threshold with the greatest accuracy for practical use in clinics.

Previous studies have used preoperative serum Tg for differential diagnosis of DTC. Petric *et al*. differentiated between follicular adenoma and carcinoma, and between follicular carcinoma and Hurthle cell carcinoma using preoperative serum Tg^18,24^. Sarah *et al*. showed that preoperative serum Tg concentration was significantly higher with initial DM. However, a cutoff for serum Tg was not presented^22,25^. Some studies found that preoperative serum Tg concentrations are not useful for differential diagnosis of DTC^15,19^. The reason for this may be the many factors that affect preoperative serum Tg concentration^20^. Therefore, in this study, we analyzed factors affecting preoperative Tg and set an optimal cutoff for preoperative serum Tg in situations such as initial DM in which Tg concentration is unequivocally raised due to a high tumor burden.

In our study, tumor histology, tumor size, blood vessel invasion, LNM > 5 and initial DM were significant factors that increased preoperative serum Tg concentration (Table 2). Among these factors, initial DM was the most significant for increased preoperative serum Tg. In multivariate linear regression analysis, the *β* value for initial DM was 3.1 times higher than for tumor histology and 42.7 times higher than for tumor size. Based on ROC curves, the preoperative Tg concentration that predicted initial DM was 63.4 ng/ml. In multivariate logistic regression analysis, the odds ratio for a Tg cutoff of 63.4 ng/ml as a predictor of initial DM was 24.62 and the effect size was significantly higher than for age at diagnosis, tumor size, blood vessel invasion, resection margin or LNM. Therefore, preoperative serum Tg may be a strong indicator of initial DM from DTC. If the preoperative serum Tg cutoff of 63.4 ng/ml were applied, 84.2% (48/57) of initial DM patients in our study could have been found at initial workup. If preoperative serum Tg is exceedingly high, clinicians should discuss with patients the possibility of bone metastasis from DTC and appropriate imaging studies in addition to chest CT scans should be pursued.

We found some discrepancies between preoperative serum Tg concentration and initial DM. When reviewing 9 patients with initial DM but preoperative serum Tg lower than 63.4 ng/ml, 7 had micronodular lung metastasis, 1 had macronodular lung metastasis and 1 had combined metastasis. These 9 patients were all PTC and had no blood vessel invasion. Of these patients, 1 patient who had combined metastasis and 1 patient with micronodular lung metastasis did not respond to radioactive iodine (RAI) therapy and died. Since these 2 patients showed a lack of RAI avidity in the metastatic lesions, we speculated that the patients did not develop an elevated Tg because of tumor dedifferentiation. Another 6 patients with micronodular lung metastasis entered remission or maintained a stable disease status after RAI therapy. They did not develop an elevated Tg. This can be attributed to a subclinical burden of microscopic metastases, which was insufficient to produce a significant amount of Tg. Also, the remaining patient with macronodular lung metastasis had a solitary lesion. Therefore, the authors speculate that if the burden of metastasis is too small or localized in a solitary site, serum Tg may not rise to sufficient levels to promote suspicion of initial DM.

In addition, in the non-DM group, we reviewed 34 patients with preoperative serum Tg who had a preoperative serum Tg greater than 1000 ng/ml. One had a large goiter, one had a multifocal PTC of more than 10, and one had a coexisting 8 cm follicular adenoma other than PTC. The remaining 31 patients had hypervascular tumors that were large enough to occupy the thyroid gland.

Savvy practitioners may use an alternative Tg cutoff derived from LRs. When preoperative serum Tg was more than 81.2 ng/ml, positive LR was 10.05, which was above 10 for the first time (sensitivity 75.4% and specificity 92.5%). NPV was 95.9% and PPV was 7.4%. A positive LR greater than 10 means that a positive test is good at ruling in a diagnosis^26^. NPV was high due to a low prevalence of initial DM. Therefore, it is also a good idea to make conservative decisions using a cutoff based on higher preoperative serum Tg (LR > 10) instead of an accuracy-derived cutoff^27^.

The strength of our study is the relatively large number of patients with initial DM^28^. Also, we proposed an optimal preoperative serum Tg concentration cutoff for practical use in clinics. In addition, we presented clinicopathological factors that may lead to elevation of serum Tg levels of DTC patients in the preoperative state. A limitation of our study was the retrospective design. Also, although our study included a relatively large number of patients with initial DM, patients were recruited from a single teaching hospital and the population was therefore prone to selection bias. Because of the low prevalence of initial DM in DTC patients, a multicenter prospective study may be needed to validate the generalizability of our findings.

In conclusion, preoperative serum Tg concentration is a useful marker for predicting the presence of initial DM from DTC, despite the presence of other interfering factors. Measurement of serum Tg in patients with biopsy-proven DTC may guide preoperative staging evaluation and initial treatment.

## Patients and Methods

The Institutional Review Board at Samsung Medical Center approved this study (IRB File No. 2017-02-056). We retrospectively screened a prospectively maintained institutional thyroid cancer database of patients who were diagnosed with and treated for DTC at Samsung Medical Center between 1994 and 2016. The final cohort was 4,735 DTC patients whose preoperative serum Tg concentrations were available for analysis. Patients were a median of 47.0 years old with 78.4% women. Total thyroidectomy was performed on 77.1% and RAItherapy was given to 64.6%. PTC was diagnosed in 97.6% and FTC in 2.4% of patients. Mean follow-up was 7.0 years. Pathological T stage was T1 in 41.9%, T2 in 2.9%, T3 in 51.6%, and T4 in 3.6%. Pathological N stage was 31.9% N1a and 9.7% N1b. Of the patients, 57 were diagnosed with initial DM. Initial DM was defined as a suspicious lesion on pathology and/or imaging such as a whole body scan, computed tomography (CT), magnetic resonance imaging, or positron emission tomography before surgery or within 6 months after surgery. In cases of ambiguity on imaging, a DM diagnosis was established by medical record review up to 6 months after surgery. For analysis, we classified patients according to site, size and number of initial DMs. First, DM was classified as lung or bone. Lung metastases were subdivided into micronodular lung metastases smaller than 1 cm and macronodular lung metastases larger than 1 cm on image study. Bone metastases were classified as solitary or multiple bone metastases. DM involving more than two organs was classified as combined metastasis. In our study, initial DM was not diagnosed at sites other than lung and bone^5,23,28^.

Serum Tg concentration was measured using immunoradiometric assays with a BRAHMS Tg plus RIA assay (BRAHMS, Henningsdoft, Germany). The functional sensitivity of the Tg plus RIA assay was 0.2 ng/ml, and analytical sensitivity was 0.008 ng/ml. Intraassay coefficient of variation (CV) was 3.4% and interassay CV was 4.9%^29^. Several factors were included in the analyses to evaluate relationships with serum Tg concentration. Age at diagnosis and sex were included as patient factors. Tumor size, multifocal tumor (tumor number), LNM, lymphatic invasion, vessel invasion, ETE, and resection margin were tumor factors. The patients with anti-Tg antibodies positive (>100 IU/mL) were excluded from the study (three patients in the initial DM group were excluded).

### Statistical analysis

Continuous data were expressed as mean ± standard deviation (SD). Data on categorical characteristics were expressed as percent values or absolute numbers. Preoperative Tg concentration was presented as median (range). For comparisons of clinical and pathological characteristics between the initial DM and non-DM groups, the χ^2^-test or Fisher’s exact test were used for categorical data, and *t*-tests or the Mann-Whitney test were used for continuous data. Multivariate linear regression analysis was used to analyze factors that affected preoperative serum Tg elevation. Receiver operative characteristic (ROC) curve analysis was used to determine the cutoff level for preoperative serum Tg to distinguish patients with initial DM. Sensitivity, specificity, and positive and negative LRs were calculated using an established method^26,30^. Pretest probability was 1.2% based on the average incidence of initial DM in the general population and in our data^1^. LRs represented how many times more (or less) frequently patients with the disease presented with a particular result compared to patients without the disease. LRs > 10 or < 0.1 were considered strong evidence to confirm or exclude initial DM, respectively. *P* < 0.05 was considered significant. Statistical analysis was performed using SPSS software version 23 (SPSS Inc., Chicago, IL, USA).

### Data availability

The datasets generated during and/or analyzed during the current study are available from the corresponding author on reasonable request.
